# Evodiamine Induces Apoptosis and Enhances TRAIL-Induced Apoptosis in Human Bladder Cancer Cells through mTOR/S6K1-Mediated Downregulation of Mcl-1

**DOI:** 10.3390/ijms15023154

**Published:** 2014-02-21

**Authors:** Tao Zhang, Shanna Qu, Qi Shi, Dalin He, Xunbo Jin

**Affiliations:** 1 Minimally Invasive Urology Center, Provincial Hospital Affiliated to Shandong University, Jinan 250021, China; E-Mail: chris2111@163.com; 2 School of Medicine, Shandong University, Jinan 250012, China; E-Mail: qushanna@sdu.edu.cn; 3 Department of Urology, the First Hospital of Xi’an Jiaotong University, Xi’an 710061, China; E-Mails: sq_880702@163.com (Q.S.); hedl_xjtu@sina.com (D.H.)

**Keywords:** evodiamine, TRAIL, Mcl-1, mTOR, S6K1, bladder cancer

## Abstract

The tumor necrosis factor-related apoptosis-inducing ligand (TRAIL), either alone or in combination with other anti-cancer agents, has been considered as a new strategy for anti-cancer therapy. In this study, we demonstrated that evodiamine, a quinolone alkaloid isolated from the fruit of *Evodia fructus*, induced apoptosis and enhanced TRAIL-induced apoptosis in human bladder cancer cells. To elucidate the underlying mechanism, we found that evodiamine significantly reduced the protein levels of Mcl-1 in 253J and T24 bladder cancer cells, and overexpression of this molecule attenuated the apoptosis induced by evodiamine alone, or in combination with TRAIL. Further experiments revealed that evodiamine did not affect the mRNA level, proteasomal degradation and protein stability of Mcl-1. On the other hand, evodiamine inhibited the mTOR/S6K1 pathway, which usually regulates protein translation; moreover, knockdown of S6K1 with small interfering RNA (siRNA) effectively reduced Mcl-1 levels, indicating evodiamine downregulates c-FLIP through inhibition of mTOR/S6K1 pathway. Taken together, our results indicate that evodiamine induces apoptosis and enhances TRAIL-induced apoptosis possibly through mTOR/S6K1-mediated downregulation of Mcl-1; furthermore, these findings provide a rationale for the combined application of evodiamine with TRAIL in the treatment of bladder cancer.

## 1. Introduction

Bladder cancer is the most common malignancy of the urinary tract and has a high rate of tumor recurrence [1]. Over 70% of the patients are non-muscle invasive bladder cancer (NMIBC) at initial diagnosis, which would be managed with transurethral resection of the tumors followed by intravesical instillation of anti-cancer agents [2]. However, up to 70% of these cases will develop recurrent tumors, with approximately 25% showing progression to a higher stage or grade [3]. *Bacillus* Calmette-Guérin (BCG) is generally considered to be the most effective intravesical agent, but the strong adverse reactions and limited response rate remain major clinical problems [4,5]. Therefore, novel intravesical agents with fewer side effects are urgently required for the treatment of bladder cancer.

Tumor necrosis factor (TNF)-related apoptosis-inducing ligand (TRAIL), a member of the TNF superfamily, has been considered to be a potential cancer therapeutic agent due to its ability to preferentially induce apoptosis in malignant cells but not in most normal cells [6]. Currently, recombinant human TRAIL is being tested in phase I clinical trials [7]. Interestingly, TRAIL has been identified as the effector molecule in intravesical BCG immunotherapy [8] and TRAIL-based therapeutics have exhibited high therapeutic potential against bladder cancer cells *in vitro* [9,10], suggesting that TRAIL could be a suitable intravesical agent for the treatment of bladder cancer. However, recent studies have demonstrated that many types of cancer cells, including certain bladder cancer cell lines, are intrinsically insensitive to TRAIL [11]. Therefore, TRAIL alone may not be sufficient for these cancer cells and the identification of effective sensitizers for TRAIL-induced apoptosis is an important issue for the development of novel cancer therapies.

**Figure 1.**
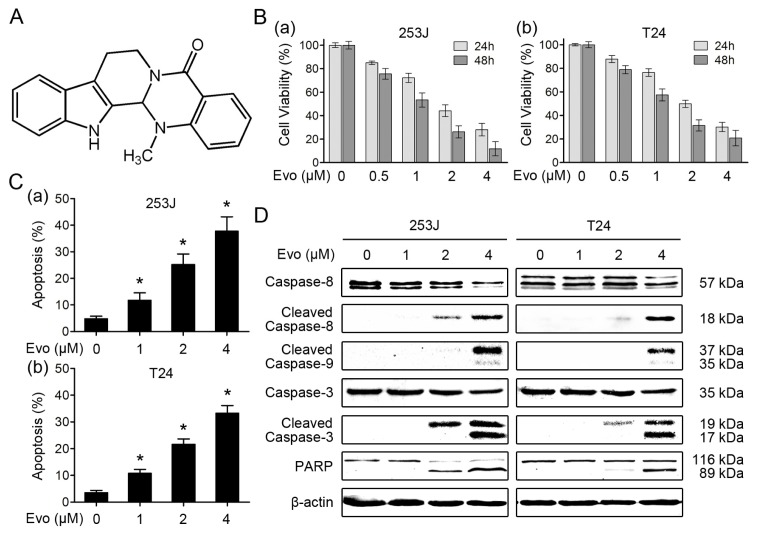
Evodiamine induces apoptosis in human bladder cancer cells. (**A**) Chemical structure of evodiamine (Evo); (**B**) Evodiamine inhibits the proliferation of bladder cancer cells. 253J (**a**) and T24 (**b**) cells were seeded in 96-well cell culture plates and treated the next day with evodiamine at the indicated concentrations for 24 and 48 h. Cell viability was assessed by MTT assay and expressed as percent of control. Columns, means of triplicate determinations; Bars, SDs; (**C**) Evodiamine induces apoptosis in bladder cancer cells. 253J (**a**) and T24 (**b**) cells were treated with evodiamine at the indicated concentrations for 24 h, and then harvested for detection of apoptosis by Annexin V staining. * *p* < 0.05 *versus* control; (**D**) Activation of caspases and PARP in apoptosis induced by evodiamine. After treatment as in panel C, cells were harvested for Western blotting to detect the cleavage of caspase-8, caspase-9, caspase-3 and PARP. β-actin was used as a loading control. Data shown are representative of three independent experiments.

Naturally occurring compounds extracted from traditional Chinese herbs have been regarded as potential anti-cancer remedies and new adjuvants to enhance the efficacy of chemotherapeutic agents [12,13]. One such promising compound is evodiamine (Figure 1A), a quinolone alkaloid that is isolated from the fruit of *Evodia fructus* [14]. Recent studies have demonstrated that evodiamine has anti-cancer activity in various tumor cells, including breast cancer cells [15], prostate cancer cells [16], colon cancer cells [17,18] and melanoma cells [19,20]. Although the precise mechanisms are not entirely elucidated, induction of apoptosis is believed to be one of the major mechanisms of action for evodiamine against cancer cells. Multiple targets, including Bcl-2, p53, and phosphatidylinositol-3 kinase (PI3K)/Akt pathway [21], have been implicated in the apoptosis-inducing effects of evodiamine. Moreover, recent studies revealed that evodiamine effectively potentiated the chemotherapeutics-induced cytotoxicity *in vitro* and *in vivo* [22,23], suggesting that evodiamine may be useful as an anti-cancer drug either alone or as an adjuvant in combination therapy.

In the present study, we explored the effects of evodiamine on human bladder cancer cells, and particularly investigated whether this agent could enhance TRAIL-induced apoptosis. We further examined the role of Mcl-1 in the evodiamine-mediated apoptosis and enhancement of TRAIL-induced apoptosis, and identified whether mTOR/S6K1 inhibition was involved in these processes. Finally, we found that evodiamine may serve as an adjuvant of TRAIL-based intravesical therapy for bladder cancer.

## 2. Results

### 2.1. Evodiamine Induces Apoptosis in Human Bladder Cancer Cells

We first investigated the effects of evodiamine as a single agent on the proliferation of human bladder cancer cells. 253J and T24 cells were treated with different concentrations of evodiamine for 24 and 48 h. As shown Figure 1B, evodiamine significantly decreased the cell viability in a dose- and time-dependent manner in both 253J and T24 cells. The half-maximal inhibitory concentration (IC50) values of evodiamine on 253J and T24 cells at 24 h were 1.90 ± 0.31 and 2.14 ± 0.26 μM, respectively. In apoptotic assays, we found that evodiamine effectively increased the percentage of Annexin V-positive cells in both bladder cancer cells (Figure 1C). Consistently, western blot analysis demonstrated that evodiamine induced the cleavage of caspase-8, caspase-9, caspase-3 and PARP (Figure 1D). Taken together, these results indicate that evodiamine induces apoptosis in human bladder cancer cells.

### 2.2. Evodiamine Enhances TRAIL-Induced Apoptosis in Human Bladder Cancer Cells

**Figure 2.**
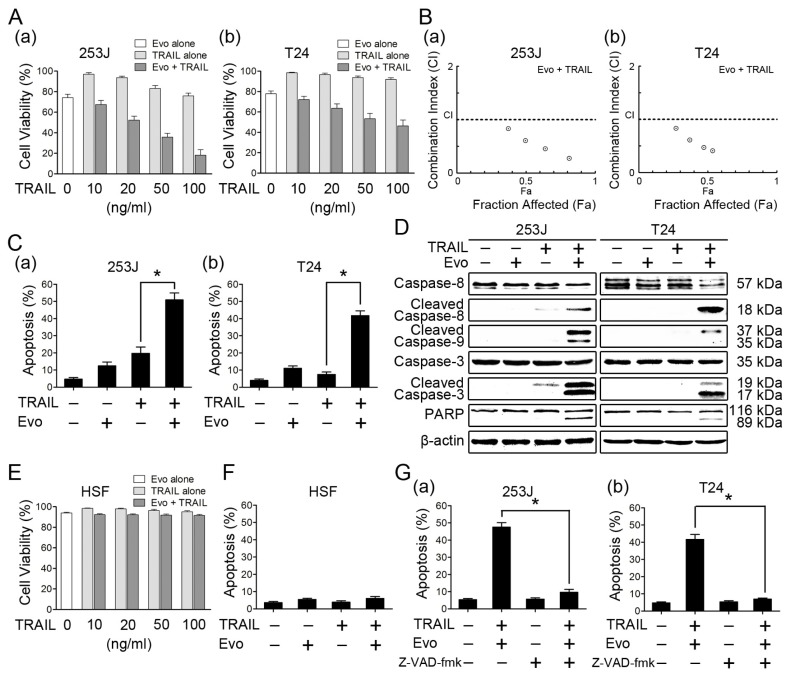
Evodiamine enhances TRAIL-induced apoptosis in human bladder cancer cells. (**A**) Evodiamine (Evo) potentiates the TRAIL-induced cytotoxicity in bladder cancer cells. 253J (**a**) and T24 (**b**) cells were seeded in 96-well cell culture plates and treated the next day with TRAIL at the indicated concentrations, with or without 1 μM evodiamine for 24 h. Cell viability was assessed by MTT assay. Columns, means of triplicate determinations; Bars, SDs; (**B**) Combinations index (CI) for interaction between evodiamine and TRAIL in bladder cancer cells. The CI was calculated based on the data presented in panel A using the CompuSyn software; (**C**) Evodiamine enhances TRAIL-induced apoptosis in bladder cancer cells. 253J (**a**) and T24 (**b**) cells were treated with 1 μM evodiamine alone, 100 ng/mL TRAIL alone, and their combination. After 24 h, the cells were harvested for detection of apoptosis by Annexin V staining. Columns, means of triplicate determinations; Bars, SDs. * *p* < 0.05; (**D**) Activation of caspases and PARP in apoptosis induced by the evodiamine and TRAIL combination. After treatment as in panel C, cells were harvested for Western blotting to detect the cleavage of caspase-8, caspase-9, caspase-3 and PARP. β-actin was used as a loading control. Data shown are representative of three independent experiments; (**E**) The cytotoxic effects of the combination treatment with evodiamine and TRAIL on normal human skin fibroblast (HSF) cells. HSF cells were treated with TRAIL at the indicated concentrations, with or without 1 μM evodiamine for 24 h. Columns, means of triplicate determinations; Bars, SDs; (**F**) The pro-apoptotic effects of the combination treatment with evodiamine and TRAIL on HSF cells. HSF cells were treated with 1 μM evodiamine alone, 100 ng/mL TRAIL alone, and their combination for 24 h. The cells were harvested for detection of apoptosis by Annexin V staining. Columns, means of triplicate determinations; Bars, SDs. (**G**) Effects of Z-VAD-fmk on apoptosis induced by the evodiamine and TRAIL combination. The given cell lines were incubated with 20 μM Z-VAD-fmk or solvent for one hour before treatment with evodiamine (1 μM) and TRAIL (100 ng/mL) for 24 h. The cells were then harvested for detection of apoptosis by Annexin V staining. Columns, means of triplicate determinations; Bars, SDs. * *p* < 0.05.

We next determined whether evodiamine could enhance TRAIL-induced apoptosis in bladder cancer cells. Evodiamine alone (1 μM), TRAIL alone (10, 20, 50, 100 ng/mL), and then the combination of evodiamine and TRAIL were applied to 253J and T24 cells. As shown in Figure 2A, 253J cells were partially resistant and T24 cells were highly refractory to TRAIL, which were consistent with previous studies [24,25]. Evodiamine at 1 μM decreased the cell viability of 253J and T24 cells by 25.80% and 22.10% respectively, but the combination of evodiamine and TRAIL exhibited much greater potency than either agent alone in decreasing the cell viability of these two bladder cancer cells. The combination indexes for these combinations were lower than 1 in both 253J and T24 cells (Figure 2B), indicating that the combination of evodiamine and TRAIL synergistically decreases the survival of bladder cancer cells. Consistently, the combination of evodiamine (1 μM) and TRAIL (100 ng/mL) was more effective than either agent alone in inducing apoptosis, evaluated by Annexin V assay (Figure 2C). Taking the 253J cell line as an example, there were approximately 12.42% and 19.71% of apoptotic cells in cells treated with evodiamine and TRAIL respectively, but 50.92% of apoptotic cells after exposure to the combination of evodiamine and TRAIL. In western blot analysis, the combination treatment was also more potent than either agent alone in inducing cleavage of caspase-8, caspase-9, caspase-3, and PARP, evidenced by either reduction of proforms or appearance of the cleaved bands (Figure 2D). These results collectively indicate that evodiamine enhances TRAIL-induced apoptosis in bladder cancer cells. In contrast, the combination treatment of evodiamine and TRAIL had no significant cytotoxic and pro-apoptotic effects on normal human skin fibroblast (HSF) cells (Figure 2E,F), suggesting that this combined treatment may be preferentially toxic to bladder cancer cells over normal fibroblast cells. We also examined the importance of caspases in evodiamine-mediated enhancement of TRAIL-induced apoptosis. Pretreatment with the pan caspase inhibitor z-VAD-fmk substantially prevented the apoptosis induced by the combined treatment (Figure 2G), indicating that the combination of evodiamine and TRAIL induces caspase-dependent apoptosis.

### 2.3. Evodiamine Reduces the Levels of Mcl-1 and Blocks TRAIL-Induced Mcl-1 Upregulation

**Figure 3.**
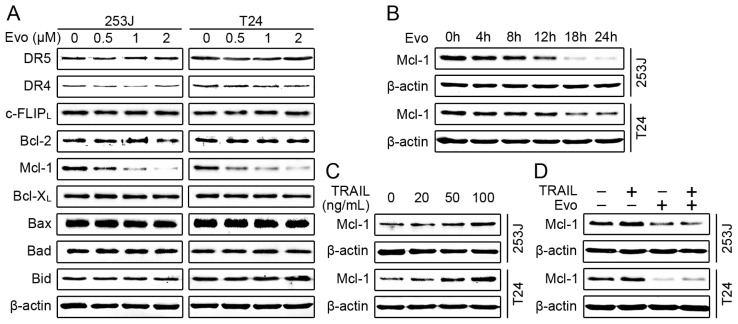
Evodiamine reduces the levels of Mcl-1 and blocks TRAIL-induced Mcl-1 upregulation. (**A**) Effects of evodiamine (Evo) on the anti-apoptotic/pro-apoptotic Bcl-2 family member proteins and the key components of TRAIL signaling pathway. 253J and T24 cells were treated with evodiamine at the indicated concentrations for 24 h, and then harvested for Western blotting to detect the indicated proteins; (**B**) Evodiamine reduces the levels of Mcl-1 in a time-dependent manner. The given cell lines were treated with 1 μM evodiamine for the indicated times and then harvested for Western blotting to detect the levels of Mcl-1; (**C**) TRAIL increases the expression levels of Mcl-1. Cells were treated with TRAIL at the indicated concentrations for 24 h and then harvested for Western blotting to detect Mcl-1 protein levels; (**D**) Evodiamine blocks TRAIL-induced Mcl-1 upregulation. The given cell lines were treated with 1 μM evodiamine alone, 100 ng/mL TRAIL alone, and their combination for 24 h. The cells were then harvested for Western blotting to detect the levels of Mcl-1. β-actin was used as a loading control. Data shown are representative of three independent experiments.

To reveal the mechanism by which evodiamine induces apoptosis and enhances TRAIL-induced apoptosis, we first examined the modulatory effects of evodiamine on DR4, DR5 and c-FLIP, which are the key components of extrinsic apoptotic pathway and often involved in drug-mediated enhancement of TRAIL-induced apoptosis [26]. Both 253J and T24 cells were treated with different concentrations of evodiamine for 24 h and harvested to detect these molecules. As shown in Figure 3A, evodiamine did not alter the levels of DR4, DR5, and c-FLIP_L_. Another well studied c-FLIP isoform, c-FLIP_S_, was hardly detectable in these two bladder cancer cell lines. We further investigated whether evodiamine affected the levels of the Bcl-2 family members (e.g., Bax, Bcl-2, Bcl-X_L_ and Mcl-1), which have been implicated in the regulation of intrinsic apoptotic pathway and cancer cell response to TRAIL-induced apoptosis [11,27]. Data in Figure 3A revealed that evodiamine did not alter the levels of Bax, Bad and Bid. Bcl-2 levels were slightly decreased in 253 cells but not in T24 cells, whereas Bcl-X_L_ levels were not reduced in both cell lines. On the other hand, the levels of Mcl-1 were significantly reduced by evodiamine in a dose-dependent manner in 253J and T24 cells. Time course analysis demonstrated that Mcl-1 reduction occurred at about 4 h and was sustained up to 24 h in both bladder cancer cells after evodiamine treatment (Figure 3B). These results indicate that downregulation of Mcl-1 may be critical for evodiamine to induce apoptosis and enhance TRAIL-induced apoptosis. Recent studies have reported that TRAIL could upregulate Mcl-1 protein levels, which is associated with TRAIL resistance [28,29]. We also observed that TRAIL increased Mcl-1 levels in a dose-dependent manner in 253J and T24 cells (Figure 3C). Moreover, Mcl-1 elevation induced by TRAIL was prevented in the presence of evodiamine (Figure 3D). Taken together, these results further support the notion that downregulation of Mcl-1 may be the underlying mechanism by which evodiamine induces apoptosis and enhances TRAIL-induced apoptosis. Therefore, we focused our further analyses on Mcl-1 in the following experiments.

### 2.4. Overexpression of Mcl-1 Attenuates the Effects of Evodiamine Alone or in Combination with TRAIL on Induction of Apoptosis

**Figure 4.**
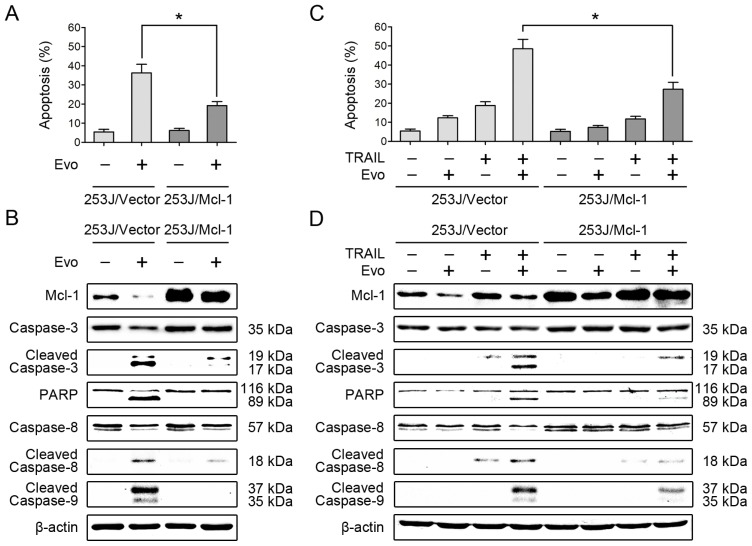
Overexpression of Mcl-1 attenuates the effects of evodiamine alone or in combination with TRAIL on induction of apoptosis. (**A**) Overexpression of Mcl-1 protects cells from evodiamine (Evo)-induced apoptosis. 253J/vector and 253J/Mcl-1 cells were treated with 4 μM evodiamine for 24 h and then harvested for detection of apoptosis by Annexin V staining. Columns, means of triplicate determinations; Bars, SDs. * *p* < 0.05; (**B**) Overexpression of Mcl-1 reduces evodiamine-induced cleavage of caspases and PARP. After treatment as in panel A, the cells were harvested for Western blotting to detect the cleavage of caspase-8, caspase-9, caspase-3 and PARP; (**C**) Overexpression of Mcl-1 protects cells from apoptosis induced by the evodiamine and TRAIL combination. Cells were treated with 1 μM evodiamine alone, 100 ng/mL TRAIL alone, and their combination for 24 h. The cells were then harvested for detection of apoptosis by Annexin V staining. Columns, means of triplicate determinations; Bars, SDs. * *p* < 0.05; (**D**) Overexpression of Mcl-1 reduces the cleavage of caspases and PARP induced by the evodiamine and TRAIL combination. After treatment as in panel C, the cells were harvested for Western blotting to detect the indicated proteins. β-actin was used as a loading control. Data shown are representative of three independent experiments.

Based on the above results, we examined whether downregulation of Mcl-1 is critical for induction of apoptosis by evodiamine or evodiamine plus TRAIL. To this end, we tested the apoptosis-inducing effects of evodiamine alone or in combination with TRAIL in cell lines that overexpressing Mcl-1. As shown in Figure 4A, overexpression of Mcl-1 significantly attenuated evodiamine-induced apoptosis in 253J cells, evaluated by Annexin V assay. Consistently, we also detected reduced levels of cleaved caspase-3 and PARP in 253J/Mcl-1 cells compared with 253J/vector cells (Figure 4B), indicating that overexpression of Mcl-1 indeed attenuates the ability of evodiamine to induce apoptosis. We further examined whether the downregulation of Mcl-1 is important for evodiamine to enhance TRAIL-induced apoptosis. The combination of evodiamine and TRAIL, as shown above, was much more potent than either single agent in increasing Annexin V-positive cell numbers and in inducing cleavage of caspase-8, caspase-9, caspase-3 and PARP in 253J cells. However, these enhanced effects were substantially reduced in cells that overexpressing Mcl-1 (Figure 4C,D). Similar results were also obtained in T24 cells stably overexpressing Mcl-1 (data not shown). Thus, these results clearly suggest that downregulation of Mcl-1 contributes to evodiamine-mediated apoptosis and enhancement of TRAIL-induced apoptosis.

### 2.5. Evodiamine Downregulates Mcl-1 Expression through the mTOR/S6K1 Pathway

**Figure 5.**
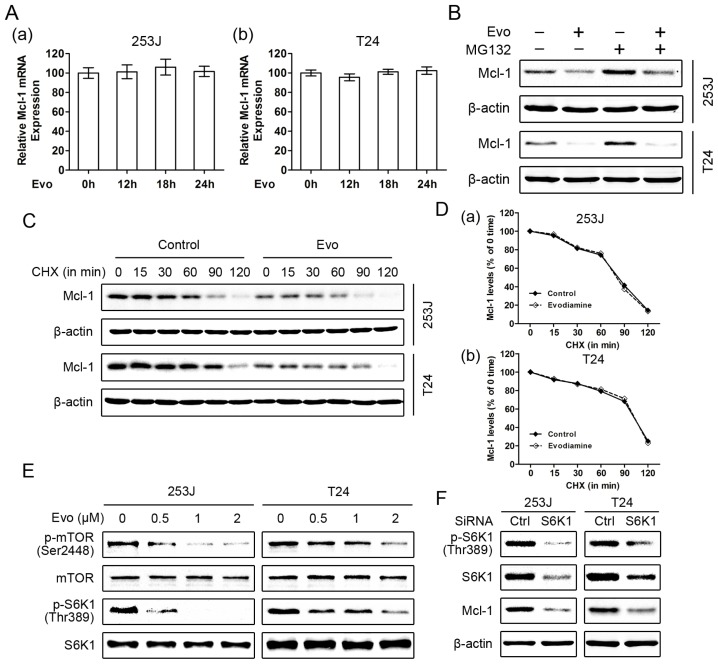
Evodiamine downregulates Mcl-1 expression through the mTOR/S6K1 pathway. (**A**) Evodiamine (Evo) does not affect Mcl-1 mRNA expression. 253J (**a**) and T24 (**b**) cells were treated with 1 μM evodiamine for the indicated times. The expression levels of Mcl-1 mRNA were analyzed by quantitative real-time PCR. Columns, means of triplicate determinations; Bars, SDs; (**B**) The proteasome inhibitor MG132 does not inhibit Mcl-1 reduction by evodiamine. The given cell lines were treated with 1 μM evodiamine for 24 h, and were either incubated or not with 20 μM MG132 for the last four hours. Cells were harvested to detect Mcl-1 protein levels; (**C**,**D**) Evodiamine does not alter the protein stability of Mcl-1. Cells were treated with or without 1 μM evodiamine for 12 h, followed by 10 μg/mL cycloheximide (CHX) for the indicated times. Mcl-1 protein levels were analyzed by Western blotting (**C**). Protein levels were then quantified with Quantity One software (Bio-Rad) and normalized to β-actin. The results were plotted as the relative Mcl-1 levels compared with those at the time 0 of CHX treatment (**D**); (**E**) Evodiamine suppresses the phosphorylation of mTOR and S6K1. 253J and RT4 cells were treated with 1 μM evodiamine for the indicated times and subsequent harvested to detect the phosphorylation of mTOR and S6K1; (**F**) Knockdown of S6K1 reduces Mcl-1 levels. Cells were transfected with control (Ctrl) or S6K1 siRNA. Forty-eight hours after transfection, the cells were harvested for Western blotting to detect the indicated proteins. β-actin was used as a loading control. Data shown are representative of three independent experiments.

To investigate the mechanisms by which evodiamine downregulates the levels of Mcl-1, we first explored whether evodiamine inhibits Mcl-1 mRNA expression in 253J and T24 cells. Both cell lines were treated with 1 μM evodiamine for different times and quantitative real-time PCR analysis demonstrated that evodiamine had no significant influence on Mcl-1 mRNA expression levels (Figure 5A), indicating that evodiamine downregulates Mcl-1 levels through a transcription-independent mechanism. We further determined whether evodiamine induces Mcl-1 degradation. Given that Mcl-1 is a rapidly turned over protein regulated by ubiquitin-mediated proteasomal degradation [30,31], we treated 253J and T24 cells with evodiamine in the absence and presence of the proteasome inhibitor MG132. As shown in Figure 5B, the presence of MG132 increased basal levels of Mcl-1, but did not prevent Mcl-1 reduction induced by evodiamine. This result suggests that evodiamine induces Mcl-1 downregulation through a proteasome-independent mechanism. We also examined whether evodiamine alters the protein stability of Mcl-1. To this end, cycloheximide (CHX) was added to cells 12 h after evodiamine treatment and the cells were harvested at the indicated time for analysis of Mcl-1 degradation rates. Even if less Mcl-1 was observed in evodiamine-treated cells, the rate of Mcl-1 clearance was not significantly affected compared with that of control cells (Figure 5C,D). Taken together, these results indicate that the decrease in Mcl-1 levels induced by evodiamine is neither due to transcriptional nor posttranslational modification. Recent studies demonstrated that Mcl-1 could also be regulated at the translational level involved an mTOR/S6K1-dependent mechanism [32,33]. Thus we evaluated the effects of evodiamine on mTOR and its downstream effector S6K1. Data in Figure 5E demonstrated that evodiamine significantly suppressed mTOR phosphorylation on Ser2448 and S6K1 phosphorylation on Thr389 in a dose-dependent manner, indicating that evodiamine effectively inhibits mTOR/S6K1 signaling. Moreover, we mimicked inhibition of mTOR/S6K1 signaling with S6K1 siRNA and then analyzed its effects on Mcl-1 expression. As shown in Figure 5F, knockdown of S6K1 effectively reduced the levels of Mcl-1, confirming that mTOR/S6K1 inhibition induces the reduction of Mcl-1. Taken together, these results suggest that evodiamine may downregulate Mcl-1 expression through the mTOR/S6K1 control of its translation.

## 3. Discussion

In the present study, we demonstrated that evodiamine could inhibit the proliferation of human bladder cancer cells and induce apoptosis. These findings were consistent with previous studies [21]. Moreover, we also showed that evodiamine effectively enhanced TRAIL-induced apoptosis in bladder cancer cells. To the best of our knowledge, this is the first study demonstrating that evodiamine could function as a sensitizer of TRAIL-induced apoptosis. Given that TRAIL plays an important role in BCG-induced anti-cancer effects [8] and TRAIL-based therapeutics have entered clinical trials [7], our findings provide a novel strategy to use evodiamine in combination with TRAIL for the treatment of bladder cancer. Thus, the current findings are of clinical significance and warrant further evaluation on the efficacy of evodiamine combined with TRAIL in animal models.

Evodiamine is the major component isolated from the fruit of *Evodia rutaecarpa* and has been shown to possess anti-cancer activity in various tumor cells. Induction of apoptosis is supposed to be the crucial factor responsible for the anti-cancer property of evodiamine, it has been shown that evodiamine could alter the balance between pro-apoptotic Bcl-2 and anti-apoptotic Bcl-2 family member proteins, and induce apoptosis by activation of either initiator caspase (caspase-9 and caspase-8) or effector caspase (caspase-3) [21]. In the present study, we found that evodiamine indeed induced apoptosis in bladder cancer cells, evidenced by Annexin V staining and Western blot analysis. Evodiamine activated caspase-9 and caspase-8 in bladder cancer cells 253J and T24, indicating that both the intrinsic and extrinsic apoptotic pathways were involved in the evodiamine-induced apoptosis. However, evodiamine did not affect the levels of Bcl-X_L_ and pro-apoptotic Bcl-2 family member proteins (e.g., Bax, Bad and Bid) in both bladder cancer cell lines, even when used at a relatively high concentration around IC50 (2 μM). It reduced the expression levels of Bcl-2 slightly in 253J cells but not in T24 cells, suggesting a cell line-dependent effect. On the other hand, evodiamine significantly reduced the levels of Mcl-1 in a dose- and time-dependent manner. Thus, it is reasonable to suggest that evodiamine altered the balance between Mcl-1 and the pro-apoptotic Bcl-2 family proteins, leading to mitochondria-mediated intrinsic apoptosis. Moreover, overexpression of Mcl-1 attenuated the ability of evodiamine to induce apoptosis, supporting the notion that the downregulation of Mcl-1 played an important role in evodiamine-induced apoptosis in bladder cancer cells. We also noted evodiamine activated caspase-8 but did not alter the expression levels of DR4, DR5 and c-FLIP. These results indicated that evodiamine-induced caspase-8 activation may be independent of the death receptor pathway. Previous studies have demonstrated that caspase-8 could also be activated by caspase-9 and caspase-3 [34,35], thus our findings in this regards are not surprising. Indeed, activation of caspase-8 induced by other chemotherapy agents (e.g., daunorubicin and camptothecin) also occured downstream from mitochondria, independently of death receptor triggering [36]. Further studies are needed to investigate the relationship between caspase-8 activation and the intrinsic mitochondrial pathway in evodiamine-treated bladder cancer cells.

Previous studies have demonstrated that the evodiamine could potentiate the chemotherapeutics-induced cytotoxicity in several tumor cells, including pancreatic cancer cells [22], breast cancer cells [23] and chronic myeloid leukemia cells [37]. Here, we found that simultaneous exposure of evodiamine and TRAIL resulted in enhanced induction of apoptosis even in TRAIL-resistant bladder cancer cell lines, but not in normal fibroblast cells. These results indicate that combined treatment with evodiamine and TRAIL may represent an attractive strategy for the safe treatment of TRAIL-resistant cancer cells without affecting normal cells. Although the detailed mechanism of TRAIL resistance is not entirely elucidated, it is probably associated with downregulation of the TRAIL receptor (DR4 and DR5) and overexpression of anti-apoptotic proteins (e.g., c-FLIP, Bcl-2 and Bcl-X_L_) [11]. Correspondingly, modulation of DR4, DR5, c-FLIP, Bcl-2 and Bcl-X_L_ is a common mechanism to enhance TRAIL-induced apoptosis. Both 253J and T24 cells have been shown to express DR4 and DR5 [24,38], however, it is unlikely to contribute to the evodiamine-mediated enhancement of TRAIL-induced apoptosis since evodiamine did not alter their expression. Previous studies also demonstrated that DR4 and DR5 receptors expressed in TRAIL-resistant bladder cancer cell lines at levels comparable to the TRAIL-sensitive ones [24], indicating that the TRAIL resistance of bladder cancer cells may depend more on the level of intracellular signaling molecules rather than differences in receptor expression. Interestingly, evodiamine alone did not significantly affect c-FLIP, Bcl-2 and Bcl-X_L_, but clearly reduced the levels of Mcl-1. Recent studies revealed that Mcl-1 could also be implicated in the regulation of cancer cell response to TRAIL-induced apoptosis [27]. Several potential anti-cancer agents, such as NVP-BKM120 (a PI3K inhibitor) and R-roscovitine (a cyclin-dependent kinase inhibitor), downregulated the levels of Mcl-1, enhancing TRAIL-induced apoptosis [39,40]. In the present study, overexpression of Mcl-1 attenuated the synergistic induction of apoptosis by the combination of evodiamine and TRAIL, suggesting that downregulation of Mcl-1 may be responsible for evodiamine-mediated enhancement of TRAIL-induced apoptosis in bladder cancer cells. Mcl-1 is primarily localized at the mitochondrial membrane where it can block the release of cytochrome c thus inhibiting the mitochondria-mediated intrinsic apoptosis [41]. We noted that overexpression of Mcl-1 attenuated activation of caspase-8 induced by evodiamine (Figure 4B) or the combination of evodiamine and TRAIL (Figure 4D), indicating that caspase-8 activation caused by evodiamine may be secondary to activation of the intrinsic apoptotic pathway. It is reasonable to suggest that evodiamine downregulates Mcl-1 expression, thus reducing its inhibitory effects on intrinsic apoptotic signaling and eventually enhancing apoptosis. During this process, caspase-8 may be further activated by caspase-9 and/or caspase-3, leading to a further amplification of apoptotic signaling. Furthermore, we can not completely exclude the possibility that Mcl-1 may have an unknown role in directly inhibiting the extrinsic apoptotic pathway, which needs further clarification. Moreover, recent studies revealed that TRAIL could upregulate Mcl-1 expression, which may have a protective effect on TRAIL-induced cytotoxicity [28,29]. In the present study, we observed that evodiamine blocked TRAIL-induced Mcl-1 upregulation in bladder cancer cells. Thus, this may also contribute to evodiamine-mediated enhancement of TRAIL-induced apoptosis.

To our knowledge, this is the first report that evodiamine could downregulate the levels of Mcl-1 in bladder cancer cells. Although Mcl-1 could be regulated at multiple levels [30,31,42], evodiamine treatment did not affect the mRNA level, proteasomal degradation and protein stability of Mcl-1, indicating that the evodiamine-mediated downregulation of Mcl-1 was neither due to transcriptional nor posttranslational modification. Previous studies demonstrated that Mcl-1 could also be regulated at the translational level [32,33]. Here, we found that evodiamine suppressed the phosphorylation of mTOR and S6K1, which were consistent with previous studies [22,43]. More importantly, knockdown of S6K1 effectively reduced the levels of Mcl-1 in bladder cancer cells, suggesting that Mcl-1 is a downstream target of S6K1. Given that mTOR is a serine/threonine kinase that functions as a critical regulator of cellular translation, and one mechanism by which mTOR regulates translation is by directly phosphorylating the key translation regulator S6K1 [44], it is convincingly concluded that evodiamine may downregulate Mcl-1 expression through the mTOR/S6K1 control of its translation. Indeed, previous study have described that inhibition of mTOR with rapamycin induced Mcl-1 but not Bcl-2 or Bcl-X_L_ block in protein translation [45]. Our data are also in agreement with recent studies showing that inhibition of S6K1 with either PF4708671 (a selective inhibitor of S6K1) or specific siRNA led to a marked decrease of Mcl-1, enhancing tamoxifen- or glucose deprivation-induced apoptosis in breast cancer cell [33,46]. Thus, inhibition of mTOR/S6K1 activity may be an effective strategy for sensitizing cancer cells to apoptotic stimuli, and mTOR/S6K1-mediated downregulation of Mcl-1 is supposed to be responsible for the evodiamine-mediated enhancement of TRAIL-induced apoptosis in bladder cancer cells. Given that several mTOR inhibitors and S6K1 inhibitors have been tested as adjuvants of chemotherapy [46,47], further study of the potential application of evodiamine and TRAIL combination in bladder cancer therapy is warranted.

## 4. Experimental Section

### 4.1. Reagents and Antibodies

Evodiamine, the proteasome inhibitor MG132 and the protein synthesis inhibitor cycloheximide (CHX) were purchased from Sigma-Aldrich (St. Louis, MO, USA). The soluble recombinant human TRAIL was purchased from PeproTech, Inc. (Rocky Hill, NJ, USA). Antibodies against Mcl-1, caspase-8, caspase-9, caspase-3, PARP, mTOR, phospho-mTOR (Ser2448), p70 S6 Kinase 1 (S6K1), phospho-S6K1 (Thr389), peroxidase-conjugated secondary antibodies were purchased from Cell Signaling Technology (Danvers, MA, USA). Antibodies against DR4, DR5, Bcl-2, Bcl-X_L_, Bax, Bad, Bid and β-actin were purchased from Santa Cruz Biotechnology (Santa Cruz, CA, USA). Antibody against c-FLIP was from Alexis Biochemicals (San Diego, CA, USA). All other reagents were purchased from Sigma-Aldrich unless otherwise specified.

### 4.2. Cell Lines and Culture Conditions

Human bladder cancer cell lines 253J and T24 were kindly provided by Dr. Leland W.K. Chung (Cedars-Sinai Medical Center, Los Angeles, CA, USA). The normal human skin fibroblast (HSF) cells were obtained from the American Type Culture Collection (ATCC, Rockville, MD, USA). Cells were cultured in Dulbecco’s Modified Eagle’s Medium (DMEM; 253J and T24) or Eagle’s Minimum Essential Medium (EMEM; HSF) supplemented with 10% of fetal bovine serum (FBS) and 1% of penicillin-streptomycin. DMEM and FBS were obtained from Invitrogen (Carlsbad, CA, USA) and EMEM was from ATCC. All cells were maintained in a humidified incubator at 37 °C with 5% CO_2_ and passaged with 0.25% trypsin-EDTA when they reached ~80% confluence.

### 4.3. Cell Viability Assay

Cells were seeded in 96-well cell culture plates and treated the next day with the given agents for the indicated times. The cells were then washed once and incubated with 0.5 mg/mL of 3-(4,5-dimethylthiazol-2-yl)-2,5-diphenyl tetrazolium bromide (MTT) at 37 °C for 4 h. Finally, the medium was discarded carefully and dimethyl sulfoxide (DMSO) was added to solubilize the formazan crystals. The absorbance was measured using the Microplate Autoreader (Bio-Tek Instruments Inc., Winooski, VT, USA) at a wavelength of 490 nm. The experiments were performed in triplicate. The combination index (CI) for drug interaction was calculated using the CompuSyn software (ComboSyn, Inc., Paramus, NJ, USA). The values of CI < 1 indicate synergistic interaction, while the values > 1 or not significantly different from 1 specify antagonistic or additive interaction, respectively.

### 4.4. Quantitative Detection of Apoptosis

Apoptosis was evaluated using an Annexin V-FITC Apoptosis Detection Kit (BD Biosciences, San Jose, CA, USA) according to the manufacturer’s instructions. Briefly, cells were treated with the indicated agents for 24 h and then harvested and washed with cold phosphate buffered saline (PBS). Data were then collected by flow cytometric analysis using a FACSCalibur (Becton Dickinson, San Jose, CA, USA). The percentage of positive cells in the upper right and lower right quadrants represent the total apoptotic cell population. For each assay, three independent experiments were conducted.

### 4.5. RNA Isolation and Quantitative Real-Time PCR

RNA was isolated using a Total RNA Extraction Kit (Fastagen, Shanghai, China) and reverse transcribed with the RevertAid™ kit (MBI Fermentas, St. Leon-Rot, Germany) according to the manufacturer’s instructions. cDNA was then subjected to a 25 μL real-time PCR carried out in a CFX96™ Real-Time PCR Detection System (Bio-Rad, Hercules, CA, USA) using the SYBR Premix Ex Taq™ II (Takara Bio, Otsu, Japan). Measurements were run in triplicates and normalized to β-actin (an internal standard) values. Primer sequences were listed as follows: Mcl-1 forward: 5′-TGCTGGAGTTGGTCGGGGAA-3′; Mcl-1 reverse: 5′-TCGTAAGGTCTCCAGCGCCT-3′; β-actin forward: 5′-GCACCACACCTTCTACAATGAG-3′; β-actin reverse: 5′-ACAGCCTGGATGGCTAC-GT-3′.

### 4.6. Plasmids and Establishment of Stable Cell Lines Overexpressing Mcl-1

Human Mcl-1 expression plasmids pcDNA 3.1-Mcl-1 was obtained from Addgene (Cambridge, MA, USA) and was verified by sequencing before transfection. Cells were transfected with the indicated plasmids, or control plasmid pcDNA 3.1 vector using Lipofectamine 2000 (Invitrogen) according to the manufacturer’s instructions. To establish stable lines that express ectopic Mcl-1, transfected cells were selected with fresh medium containing 600 μg/mL G418 (Invitrogen) for two weeks. The surviving cells were pooled as the stable cell lines and Mcl-1 expression was verified with Western blotting.

### 4.7. SiRNA and Transfection

The human S6K1 siRNA (target sequence: 5′-GGACAUGGCAGGAGUGUUU-3′) and a negative control siRNA (nonspecific target sequence: 5′-UUCUCCGAACGUGUCACGUTT-3′) were purchased from GenePharma Co. Ltd. (Shanghai, China). Transfection of siRNAs using Lipofectamine 2000 (Invitrogen) was performed according to the manufacturer’s instructions. Cells were cultured for 48 h after transfection and then harvested for further experiments.

### 4.8. Western Blot Analysis

Cells were treated with the indicated agents, and the whole cellular lysates were then prepared with RIPA buffer (50 mM Tris (pH 8.0), 150 mM NaCl, 0.1% SDS, 1% NP40 and 0.5% sodium deoxycholate) containing proteinase inhibitors, 1% cocktail and 1 mM PMSF. For immunoblot analyses, a total of 30–60 μg of protein was separated by 8%–12% SDS-PAGE and transferred to nitrocellulose membranes. The membranes were blocked with 5% skim milk in Tris Buffered Saline (TBS) for 1 h at room temperature and probed with primary antibodies overnight at 4 °C. After being washed with TBS-Tween (0.1%), the membranes were incubated with horseradish peroxidase-conjugated secondary antibody for 1 hour at room temperature. Protein signal was then detected with the ECL chemiluminescent detection system (Bio-Rad). Loading differences were normalized using a monoclonal β-actin antibody.

### 4.9. Statistical Analysis

Statistical analyses were performed using SPSS 15.0 (SPSS Inc., Chicago, IL, USA). All assays were repeated in triplicates in three independent experiments, and quantitative data were presented as mean ± SD. The differences among the control and various treatment groups were compared by one-way ANOVA, followed by Dunnett’s *t* test for separate comparisons. *p* < 0.05 was considered statistically significant.

## 5. Conclusions

In conclusion, we herein demonstrate for the first time that evodiamine induces apoptosis and enhances TRAIL-induced apoptosis in human bladder cancer cells, possibly through mTOR/S6K1-mediated downregulation of Mcl-1. Therefore, our studies highlight a potential novel strategy to use evodiamine as an adjuvant of TRAIL-based intravesical therapy for bladder cancer.
